# Impact of chronic statin-pretreatment on myocardial damage as assessed by Cardiac Magnetic Resonance findings in patients with acute ST-elevation myocardial infarction

**DOI:** 10.1186/1532-429X-14-S1-O67

**Published:** 2012-02-01

**Authors:** Georg Fuernau, Ingo Eitel, Steffen Desch, Suzanne de Waha, Gerhard Schuler, Holger Thiele

**Affiliations:** 1 Clinic for Internal Medicine/Cardiology, University of Leipzig - Heartcenter, Leipzig, Germany

## Background

Recent data have demonstrated a lower mortality in acute ST-elevation myocardial infarction (STEMI) patients with previous treatment with statins, especially in patients with high risk profiles. Moreover, a significant reduction in enzymatic infarct size in non-STEMI patients could be observed. However, systematic data of the impact of chronic statin pre-treatment on myocardial damage and reperfusion injury assessed with the gold standard cardiac magnetic resonance imaging (CMR) are lacking.

The aim of our prospective study was therefore to assess the effects of a chronic statin pre-treatment on myocardial damage as assessed by CMR in patients with acute reperfused STEMI.

## Methods

We enrolled 220 consecutive patients with acute STEMI undergoing primary PCI within 12 hours of symptom onset in our study. CMR studies were performed 2-4 days after the index eventinfarction (median 3 days, interquartile range 2, 4 days) using a standard infarction protocol.

## Results

Of 220 patients, 35 (15.9 %) had chronic statin pre-treatment. There was no difference in pain-to-balloon time between the two groups (statin vs. no statins: 300.2±178.2 vs. 288.7±179.8 min, p=0.73), but the statin group showed a trend towards a higher TIMI risk-score (4.5±2.0 vs. 3.7±2.3, p=0.054) and higher age (68.7±11.0 vs. 64.9±12.6 years, p=0.09). In cMRI no differences in the area at risk (35.8±12.9 vs. 35.6±10.9 %LV, p=0.96) and left ventricular ejection fraction (48.4±11.4 vs. 50.5±12.0 %, p=0.35) could be observed. Infarct size showed no statistical significant difference (18.0±11.5 vs. 15.3±12.0 %LV, p=0.23) but statin pre-treatment resulted in a significant higher myocardial salvage index as compared to patients without statin pre-treatment (59.8±27.4 vs. 49.1±27.0, p=0.04).

## Conclusions

Statin pretreatment in patients with STEMI is associated with a higher myocardial salvage index and may explain the recently observed improved short time survival. Larger studies are needed to conclusively ascertain our results.

## Funding

None.

